# Removal of graffiti paint from construction materials coated with TiO_2_-based photocatalysts

**DOI:** 10.1007/s11356-024-34467-4

**Published:** 2024-08-01

**Authors:** Eva Jimenez-Relinque, Francisco Jose Rubiano, Marta Castellote

**Affiliations:** 1 Department of Construction, Research Group of Sustainable Interaction of Construction Materials With the Environment, Eduardo Torroja Institute for Construction Science, IETcc (CSIC), Serrano Galvache 4, 28033 Madrid, Spain; 2 Department of Construction. Research Group of Structural Systems and Concrete, Eduardo Torroja Institute for Construction Science, IETcc (CSIC), Serrano Galvache 4, 28033 Madrid, Spain

**Keywords:** Titanium dioxide, Photocatalysis, Graffiti, Cleaning, Anti-graffiti, Construction

## Abstract

Graffiti on construction materials has significant social and economic impacts, especially on artistic and historical artefacts. Anti-graffiti protective coatings are used to generate low surface energies that limit graffiti adhesion to the surface, thereby reducing surface damage and facilitating removal. The anti-graffiti properties of three commercial TiO_2_-based coatings were tested under outdoor exposure conditions using four colours of graffiti paint (red, blue, black, and white). Chemical removers were used to clean the stained surfaces to understand the impact of the photocatalytic coatings during the conventional cleaning procedure. The effectiveness of cleaning was assessed by visual observations, colour measurements, and the percentage of residual stain. The anti-graffiti efficacy was strongly dependent on the colour of the graffiti and characteristics of the TiO_2_ coating. The cleaning performance of TiO_2_-treated samples was likely related to the photocatalytic redox reactions that decompose the graffiti. Additionally, their hydrophilicity may also prevent the adhesion and/or penetration of graffiti paint on the surface and/or pore matrix.

## Introduction

Graffiti is an ever-growing phenomenon, particularly in urban contexts. Although it is sometimes considered a form of “street art” (Masieri and Lettieri 2017, Ross 2016), when it is the result of vandalism, it has significant negative aesthetic impacts on buildings. Graffiti in cultural heritage sites, monuments, and historic buildings is not just an aesthetic problem; it is a threat to conservation efforts. In addition to economic aspects, the interaction of graffiti with the substrate, as well as removal procedures, endangers the lifetime of historical buildings (Masieri and Lettieri 2017).

Currently, graffiti removal is based on mechanical and/or chemical methods (Sanmartín et al. 2014), which can be expensive and sometimes unsatisfactory (Pozo-Antonio et al. 2018). Mechanical cleaning typically employs high-pressure water or air-abrasion techniques using fine particles. These methods can create fissures or cracks that may cause further penetration of solubilised graffiti materials into the substrate and favour the absorption/adhesion of future graffiti paints (Carmona-Quiroga et al. 2017; Pozo-Antonio et al. 2018). Chemical methods usually involve the use of organic solvents and alkali-based paint removers, which are hazardous to the operator’s health and safety (Germinario et al. 2016). Solvent-based cleaning methods may also harm the treated objects; solvents can be retained in the pore systems of porous materials, resulting in salt precipitation (Pozo-Antonio et al. 2018). Although used for graffiti removal, laser ablation techniques are costly, time-consuming, and require further refinement. (Chapman 2000, Costela et al. 2003; Pozo-Antonio et al. 2016; Rivas et al. 2012; Siano et al. 2012), they are costly, time consuming, and require refinement (Chapman 2000, Pozo-Antonio et al. 2018; Rivas et al. 2012). In addition, as with traditional cleaning methods, the cleaning efficiency depends on the type of paint to be extracted (Pozo-Antonio et al. 2018; Rivas et al. 2012), and can even cause mineralogical alterations, giving the appearance of fractures (Pouli et al. 2008; Pozo-Antonio et al. 2016, 2018).

To address these problems, anti-graffiti coatings are applied to surfaces as a preventive strategy, providing a protective barrier (preventing the paint from penetrating the substrate) and facilitating easier cleaning with solvents and/or water at low pressures (Gomes and Dionísio 2017, Pozo-Antonio et al. 2018). Two types of anti-graffiti coatings are commonly used, sacrificial and permanent. Sacrificial products are removed together with paint and must be reapplied, whereas permanent systems can withstand multiple cleaning cycles.

Since the mid-1990s, the ability of TiO_2_ to remove/decompose organic and inorganic pollutants and particulate matter has been exploited to create self-cleaning materials (fabrics (Qi and Wang 2011, Xu et al. 2015), paint (Maggos et al. 2007), glass (Hattori et al. 2001; Watanabe et al. 1999), tiles (Ke et al. 2014; São Marcos et al. 2008), and cement (Jimenez-Relinque et al. 2015; Laplaza et al. 2017)). Self-cleaning properties can be obtained by combining a substrate material with nano-TiO_2_ powder or a thin-film coating. Under UV radiation, TiO_2_ generates electron–hole pairs, which decompose substances that come into contact with them via redox reactions and prevent them from building up. This photocatalytic activity is accompanied by photo-induced hydrophilicity; this increases cleaning effectivity as water spreads over the surface rather than remaining as droplets, preventing the adhesion of organic contaminants and dust (Banerjee et al. 2015; Watanabe et al. 1999). The hydrophobicity of conventional anti-graffiti systems has been assumed to control the adhesion characteristics of graffiti paint to the substrate surface, particularly when repeated water-pressure cleaning cycles are required (Banerjee et al. 2015).

Considering the limitations of the described cleaning methods, photocatalytic self-cleaning may effectively reduce the impact of graffiti on buildings and cultural heritage sites by reducing aesthetic damage and deterioration. However, the majority of reports on such coatings have focused on the evaluation of the photo-mineralisation of coloured organic dyes as model compounds under laboratory conditions (Banerjee et al. 2015; Jimenez-Relinque and Castellote 2019a, b; Petrovič et al. 2012). This study intends to fill this gap by determining whether a preventive TiO_2_ self-cleaning treatment is effective for cleaning graffiti paints. This study was conducted within the framework of the European Life Project Life-Photoscaling (LIFE PHOTOSCALING, LIFE 13/ENV/ ES/001221, http://www.life-photoscaling.eu/). Three commercial TiO_2_ emulsion formulations were sprayed onto concrete slabs under real-world outdoor conditions. The cleaning performance of the four colours of graffiti paints was evaluated using a digital camera and image processing software (ImageJ Fiji). Next, the graffiti paints were applied, the effect of the TiO_2_ coating on the ease of cleaning with a conventional solvent and water pressure was investigated.

The influence of parameters such as UV–Vis absorbance, water capillarity absorption, hydrophobic properties, total porosity, and pore size distribution was considered. The photocatalytic efficiency of the treatments was assessed using standardised tests for self-cleaning rhodamine B (RhB) dye and nitrogen oxide (NO_*x*_) air purification.

## Experimental

### Materials

**Fig. 1:**
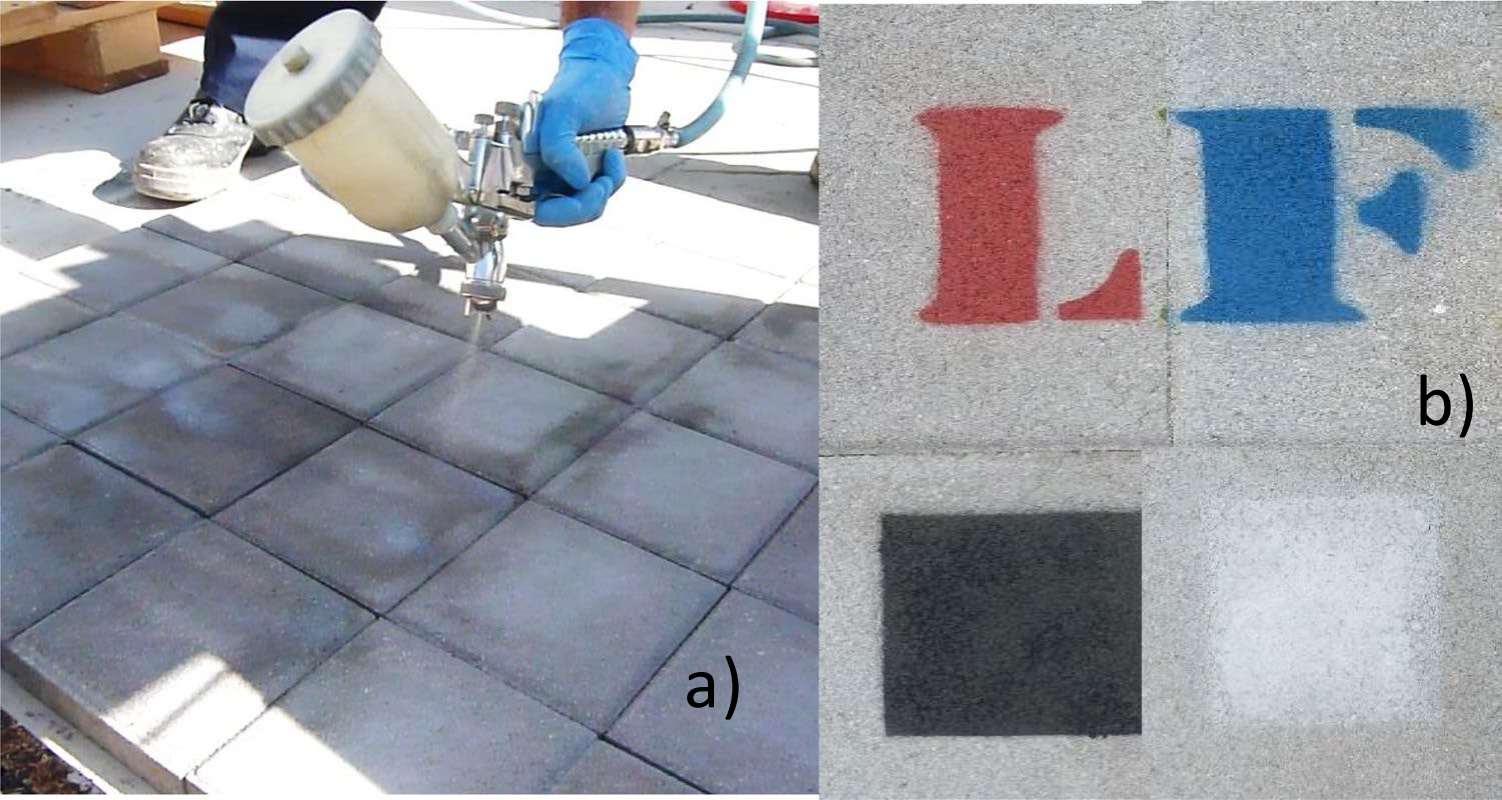
**a** Spraying of the photocatalytic coatings on the slabs. **b** Examples of the graffiti painted on the tiles

Three commercial photocatalytic emulsions (T1, T2, and T3) were sprayed onto two sets of 1 × 1 m^2^ slabs of precast concrete tiles, typically used in the streets in Madrid (Fig. 1a). An additional paving slab was left untreated as a reference (UT). All the coatings contained a TiO_2_-based photocatalyst and were applied by the manufacturers or by the staff of the IETcc, following the instructions given by the manufacturers. The compositions and characteristics of the photocatalysts cannot be disclosed for confidentiality reasons. Blue (5010), red (3000), black (9005), and white (9010) spray paints from Dupli-Colors S.L. were applied from a distance of 10 cm for 10 s on the TiO_2_-coated and untreated reference slabs (Fig. 1b). According to technical sheets, all paints were organic, though their compositions were unspecified.

### Characterization of the materials

X-ray diffraction (XRD) was used to determine the mineralogical composition of the four paints. The functional groups of the binder and pigments in each paint were identified using Fourier-transform infrared (FTIR) spectroscopy.

The UV–Vis absorption spectra of the TiO_2_ coatings were obtained using a Shimadzu UV-2600 UV/VIS/NIR spectrometer equipped with a diffuse reflectance accessory sphere (DRS).

The hydrophobicity/hydrophilicity of the samples was determined by measuring the dynamic contact angle of water droplets under dark and illuminated conditions. Irradiation was performed using two UV–Vis fluorescence tubes (Philips Actinic BL TL-D 15 W) with intensities of 356 Lux (400–850 nm) and 3.8 Wm^−2^ (315–400 nm). Droplets (volume, ~ 2 μL) were injected slowly onto the solid surface by a syringe. To eliminate the impact of droplet evaporation on the measured contact angles, images of the droplets were captured directly after their deposition (within 5 min). The photographs were processed and analysed using ImageJ 1.52n (Fiji) plugin Contact angle (Plugin Contact angle 2019). The aesthetic compatibility of the TiO_2_ photocatalyst treatments, in terms of preserving the original colour of the substrate, was determined by spectrophotometric measurements (Konica Minolta CM-2300d spectrophotometer).

**Fig. 2:**
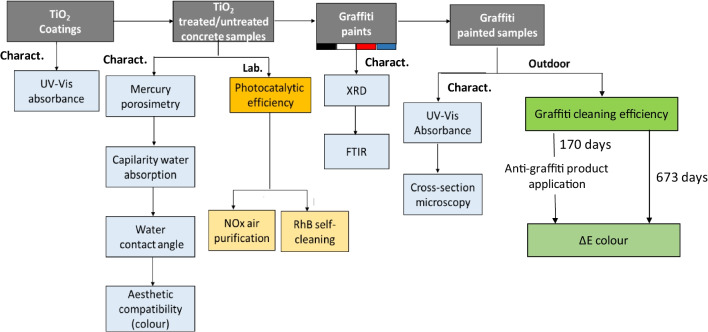
Flow chart of the experimental procedure

Initially, the graffiti-painted specimens were analysed in the laboratory using UV–Vis reflectance to assess their distribution over the surface. Optical microscopy evaluations of cross-sections of the graffiti-painted fractured specimens were also performed to determine the internal distribution of the paints in the substrate. The materials were characterised as shown in the flow chart (Fig. 2).

### Photocatalytic efficiency

The photocatalytic efficiency of the TiO_2_ coatings was analysed by two standardised laboratory methods: UNI 11259:^2008^ (degradation of the colour of rhodamine B (RhB)) and ISO 22197–1:^2007^ (removal of NO_*x*_).

RhB is commonly used to evaluate the self-cleaning properties of photocatalytic construction materials (Jimenez-Relinque et al. 2015). The standard specifies that a CIE L*a*b* colorimeter is used, by monitoring “a*” (the reference parameter for the red colour). However, the photocatalytic performance calculation using the a* (red) coordinate is only suitable for white or light cement concrete. In coloured substrates (grey cement, in our case), measurements can be distorted by the loss of RhB colour, which implies the emergence of the substrate’s original colour. Thus, in this study, the photodegradation of RhB was determined using the global colour variation (Δ*E*) according to the CIE76 formula. This calculation improves the reliability of experimental procedures for determining photocatalytic performance (Diamanti et al. 2013; Laplaza et al. 2017).

$${\Delta E}_{{\text{Lab}}^{*}}={{(\Delta L}^{*2}+{\Delta a}^{*2}+{\Delta b}^{*2})}^{1/2}{}^{*}100$$

The specimens were irradiated with UVA LEDs for 4 and 26 h (irradiance, 2 Wm^−2^). Three specimens were tested, and three measurements were performed for each specimen using a portable spectrophotometer (Konica Minolta CM-2300d).

The NO_*x*_ removal efficiency was measured according to ISO 22197–1:^2007^, with some modifications to reduce the irradiation time. It consists of introducing a NO/air mixture with a NO concentration of 1000 ± 50 ppb_v_ and gas flow 3 Lmin^−1^ into the reactor in laminar flux at relativity humidity of 50% and temperature of 25 °C. Two Philips Actinic BL 15 W/10 SLV UVA fluorescent tubes were used to irradiate the samples for 30 min. The photocatalytic activity of the material was determined from the decrease in the NO_*x*_ concentration under UV irradiation using a chemiluminescence analyser (AC-32 M, Environment S.A.).

### Outdoor monitoring

**Fig. 3:**
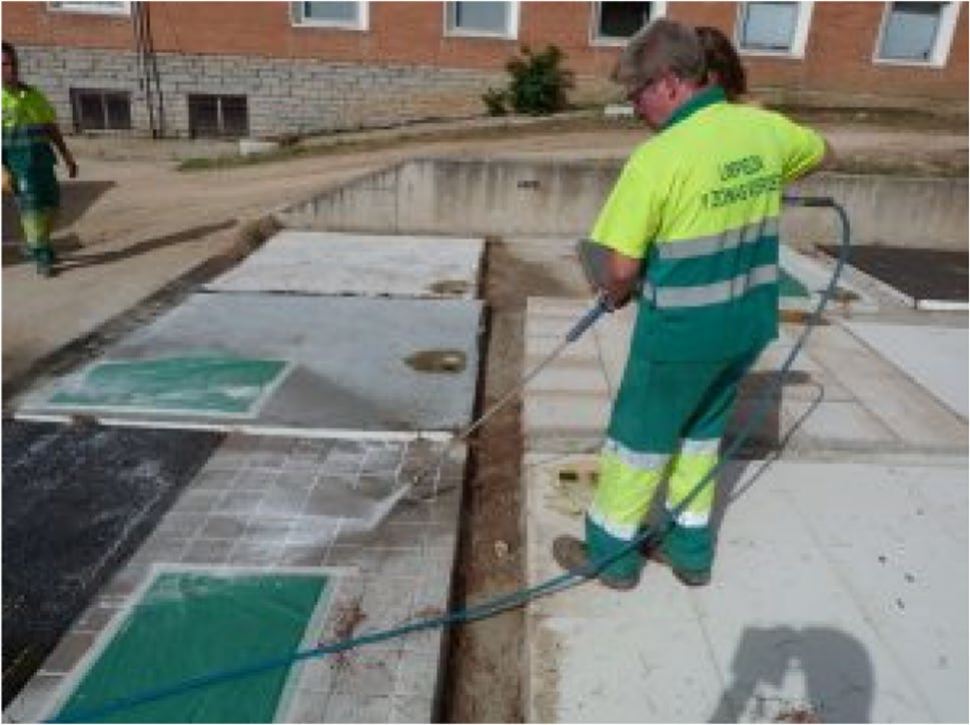
Staff of Madrid City Council cleaning the graffiti on the slabs

One set of graffiti-painted slabs was exposed to outdoor conditions for 18 months (Jun 2017 to Dec 2018), whereas the second was left outdoor just for 5.5 months (Feb 2017 to Jul 2017). After this time, in the last case, an anti-graffiti gel-stripper product (CK-Procap ECO Plus) was applied by the staff of Madrid City Council according to their regular procedure (Fig. 3). The pure gel, which sticks to the graffiti paint, was applied by brush. After 5 min, it was flushed with pressurised water for a duration of 5 min.

**Fig. 4:**
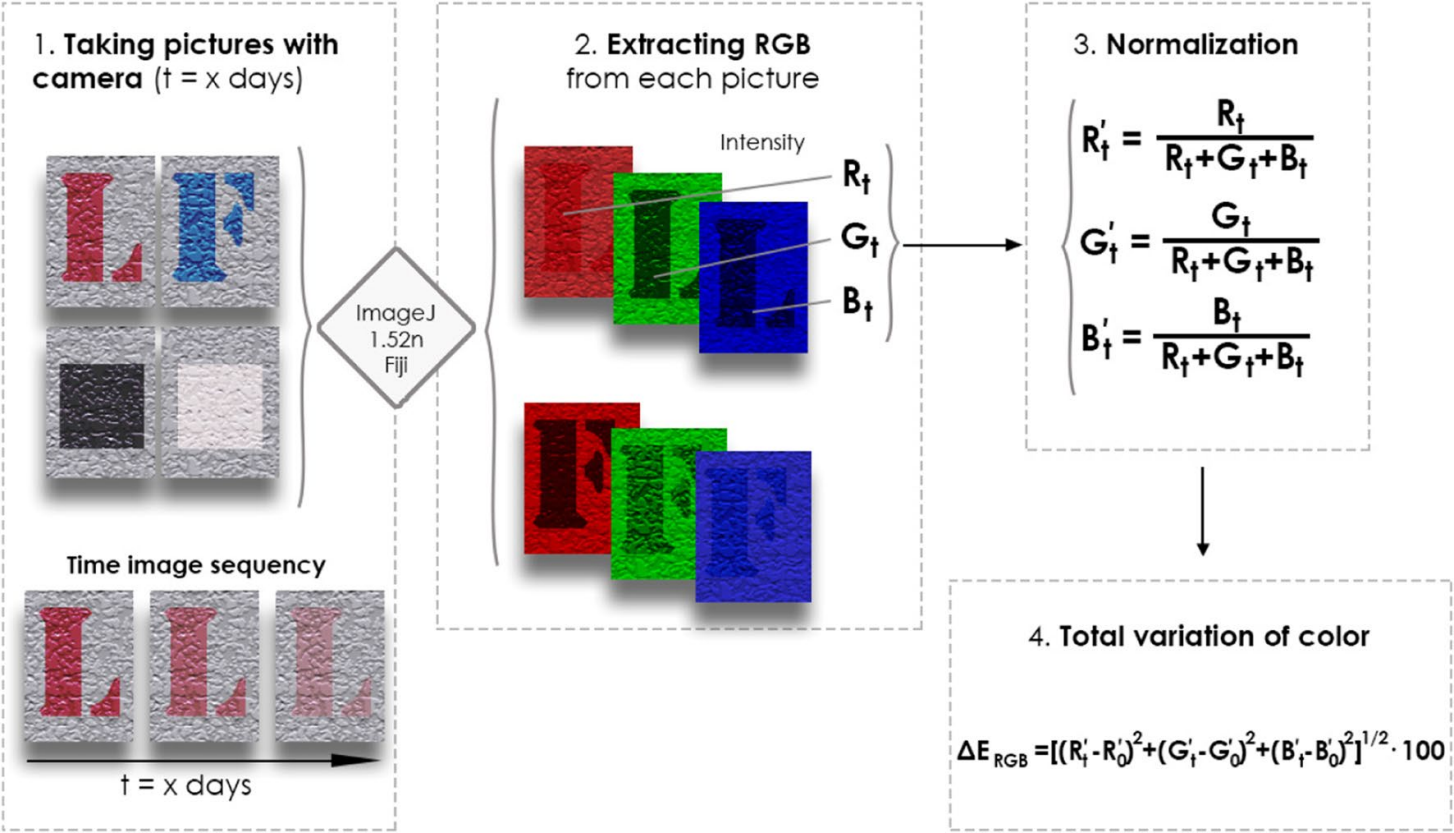
Schematic diagram of the procedure for the calculation of the variation of global colour

The graffiti paint colour intensity was monitored by changes in the surface chromatic properties during outdoor exposure, using a digital camera as an acquisition device and digital image processing software (ImageJ 1.52n, Fiji) to detect in situ RGB (red, green, and blue) colour variations. Digital images of the graffiti on all substrates were periodically captured using the digital camera. The images were imported into the ImageJ software, and the average RGB pixel intensities were collected. The analysis was performed thrice to obtain average RGB intensity values. To eliminate the potential effects of environmental conditions, such as lighting, position, and angle of the camera, the measured values of *R*, *G*, and *B* were normalised (*R´*, *G´* and *B´* values). The schematic diagram of the analytical procedure used for the calculation of global colour change (Δ*E*_RGB_) during outdoor exposure is depicted in Fig. 4.

This procedure allows analysis of the effect of the ageing of graffiti paints in situ under outdoor exposure conditions and differentiates the potential effect of TiO_2_ treatments on the time of disappearance. Only 13% of the 340 data points were outliers. These anomalies usually occur because of improper adjustment or white balance on the digital camera functionality (the picture appears bluish to the naked eye), or because the photo was taken at an angle that created shadows. Although the procedure includes post-image normalisation, these errors could not be corrected, and these data were excluded from the analysis.

Furthermore, the effectiveness of removing graffiti paint with the anti-graffiti gel-stripper product in all the slabs was evaluated based on residual paint stains by comparing the difference between the total colour of pictures taken before painting and after the cleaning.

## Results

### Characterisation of the graffiti paints

**Fig. 5:**
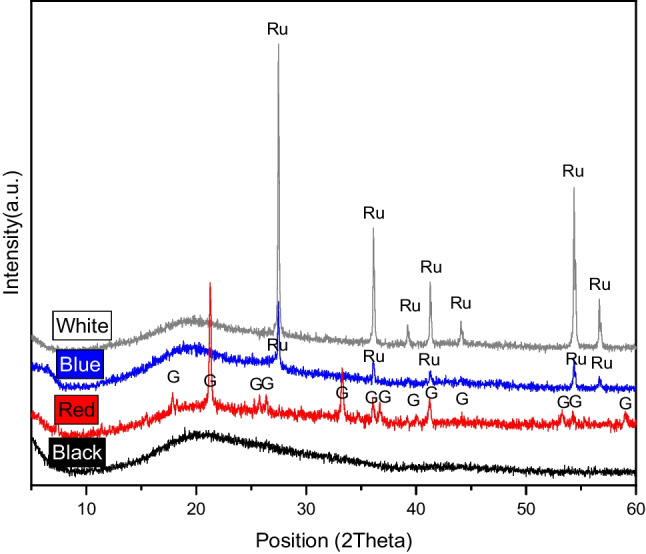
XRD patterns of the graffiti paints

The only mineral phase identified by X-ray diffraction (Fig. 5) in the white and blue paints was rutile (TiO_2_), which confers whiteness, brightness, and opacity. Only goethite (α-FeO(OH)) was detected in the red paint, whereas no crystalline phases were detected in the black paint.

**Fig. 6:**
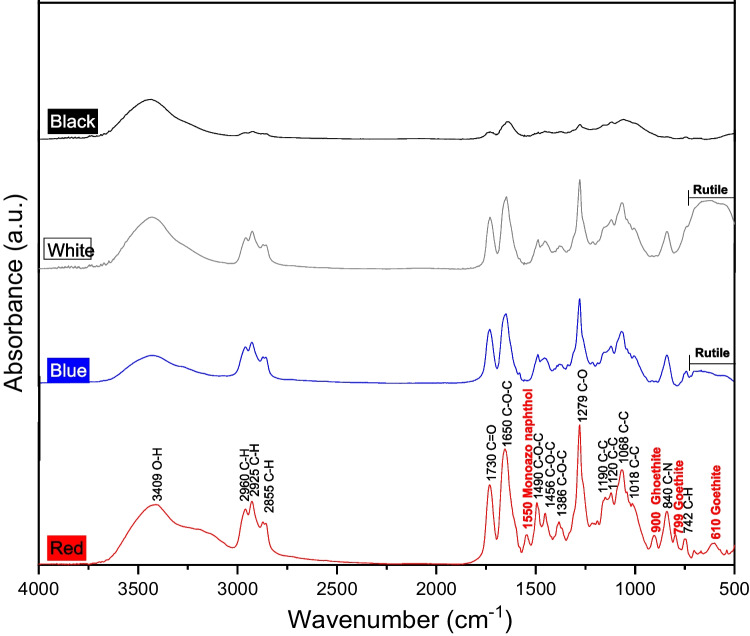
FTIR spectra of the graffiti paints

The FTIR spectra of the graffiti samples indicated that all were composed of alkyd and nitrocellulose resin binders (Fig. 6). The detected signals indicate the presence of C–H and ester functional groups. The peaks at 2960, 2925, and 2855 cm^−1^ corresponded to the C–H stretching vibrations of the alkanes. The peaks between 1730 and 1000 cm^−1^ suggested the presence of esters. The peak at 1729 cm^−1^ was attributed to the stretching vibration of C = O and those in the range of 1700–1300 cm^−1^ corresponded to the COC group stretching vibration. Characteristic peaks of the cellulose nitrate component were detected at 1279, 1068, and 840 cm^−1^, corresponding to the C–O, C–C, and C–N stretching modes, respectively. The peak at 742 cm^−1^ corresponding to the out-of-plane aromatic C–H vibration can be attributed to a minor binder modification with styrene–acrylic resins. Germinario et al. (Germinario et al. 2016) characterised the same paints by multi-analytical (pyrolysis–gas chromatography–mass spectrometry, FTIR, and µ-Raman) techniques and identified 2-ethylhexyl acrylate (2EHA) as a minor binder for the black and blue paints, and a 2EHA–styrene mixture for white and red. Additives such as di-isooctyl adipate and di-isobutyl aconitate were also detected in all the paints. The broad absorbance band at 3409 cm^−1^ arose from the O–H stretching vibration of water.

Red paint showed three additional peaks at 900, 799 and 610 cm^−1^, associated with the presence of goethite, corroborating the X-ray diffraction results. The peak detected at 1550 cm^−1^ was attributed to the monoazo naphthol AS PR170 red pigment with pyrrole C = C and aza C–N stretching (Germinario et al. 2016). Rutile, which was identified by XRD in the blue and white paints, could be identified from the very broad absorption in the low-wavenumber range (900–500 cm^−1^). In a previous study (Germinario et al. 2016), copper phthalocyanine (αCuPc) pigment was also identified in the blue paint.

### Characterisation of the substrate and TiO_2_ coatings

**Fig. 7:**
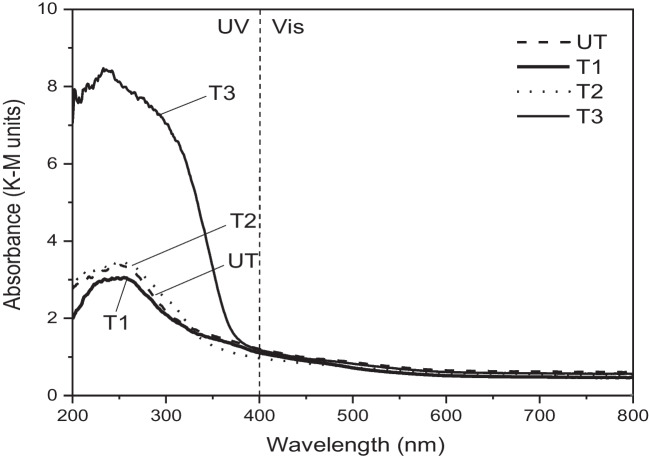
Kubelka–Munk (K–M) absorption curves of TiO_2_ coated and uncoated substrate samples

The UV–Vis absorption spectra (Fig. 7) show that all samples present higher absorbance in the UV (< 400 nm) range than in the visible light range. T1 and T2 show similar absorbance signals and band-edge positions to the uncoated sample, whereas the T3-treated sample was the most UV-absorbent and showed a blue shift in the adsorption edge.

A comparison of the colours before and after photocatalytic coating, according to the EN 15802:2010 standard, indicated that all the coatings were aesthetically compatible with the substrate (Table 1). The colour change of the concrete substrate induced by these treatments was below the perception threshold (ΔE* < 3) and the generally accepted threshold value (ΔE* ≤ 5), even for the most restrictive applications (ancient building restoration) (Munafò et al. 2015).

**Table 1:** Chromatic parameters of the substrate before (UT) and after the application of the TiO_2_ coatings (T1–T3). (L*: lightness, a*: red/green hue, b*: yellow blue hue, and Δ*Ε*_Lab_*: total colour variation)

	UT	T1	T2	T3
L*	63.64 ± 0.23	65.60 ± 1.42	62.84 ± 0.47	62.46 ± 0.18
a*	1.29 ± 0.05	0.78 ± 0.12	1.12 ± 0.08	1.17 ± 0.08
b*	6.97 ± 0.19	6.42 ± 0.39	5.37 ± 0.08	7.33 ± 0.17
∆E*	-	2.10 ± 1.21	1.80 ± 0.27	1.24 ± 0.06

**Fig. 8:**
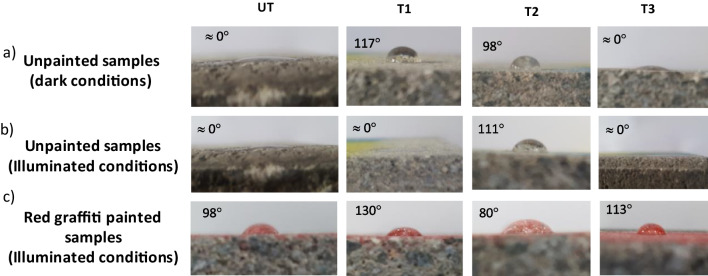
Water dynamic contact angle on the **a** unpainted samples in the dark, **b** unpainted samples under irradiation, and **c** red-painted samples under irradiation

The water dynamic contact angles on the treated and untreated surfaces without graffiti paint indicated that the unpainted substrate was hydrophilic under dark and illuminated conditions (Fig. 8). Photocatalytic coatings, T1 and T2, make it hydrophobic under dark conditions, with contact angles of 117 and 98° respectively. T1 exhibited photoinduced hydrophilicity under irradiation, whereas T2 did not. T3 showed a water-spread hydrophilic behaviour under both dark and irradiated conditions.

### Characterisation of the graffiti-painted surfaces

The water contact angle over the graffiti-painted surfaces showed a loss of hydrophilicity in all cases, regardless of the graffiti colour (see the red graffiti under illuminated conditions in Fig. 8). The hydrophobic behaviour is thought to originate from the alkyd and nitrocellulose resin binders in the graffiti paints.

**Fig. 9:**
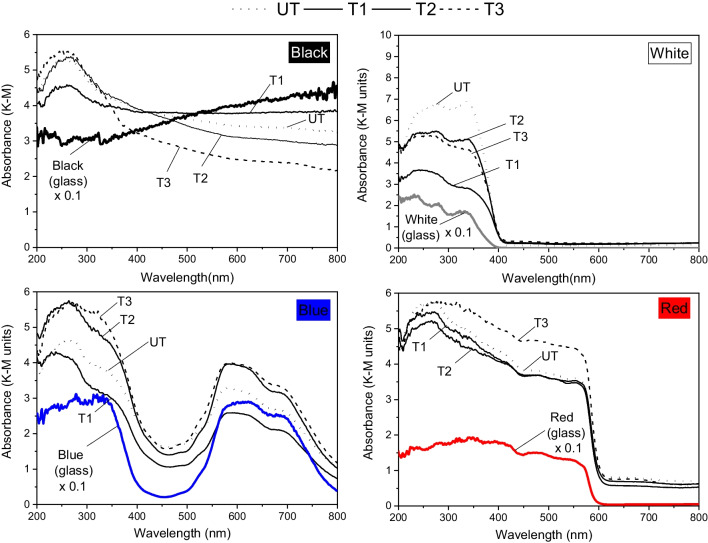
Absorption spectra (range: 200–900 nm) of the graffiti paint on glass and graffiti-painted samples (TiO_2_-treated (T1, T2, and T3); and untreated (UT))

The initial absorption spectra of the graffiti-painted laboratory specimens are shown (Fig. 9) along with the DR spectra of the paints on glass. On the graffiti-painted samples, a peak-shaped signal can still be observed in the UVA range corresponding to the underlying substrate (Fig. 7), suggesting that the graffiti paint did not entirely cover the substrate (untreated or TiO_2_-treated). Thus, in the treated materials, it is feasible that the photocatalytic redox process occurs under sunlight in the exposed parts coated by TiO_2_.

**Fig. 10:**
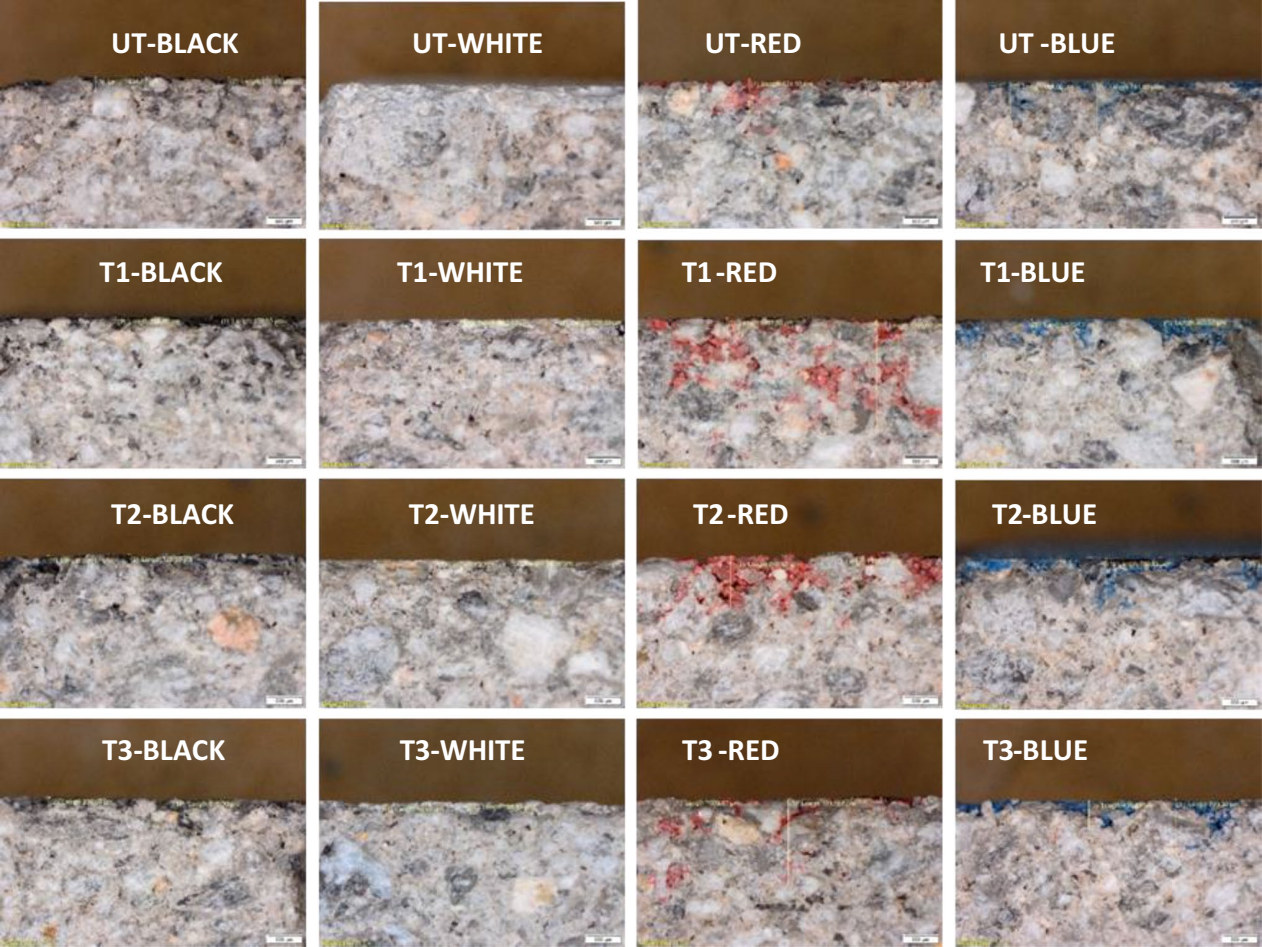
Optical micrographs of the cross-sections of the specimens painted with the graffiti (four colours)

**Fig. 11:**
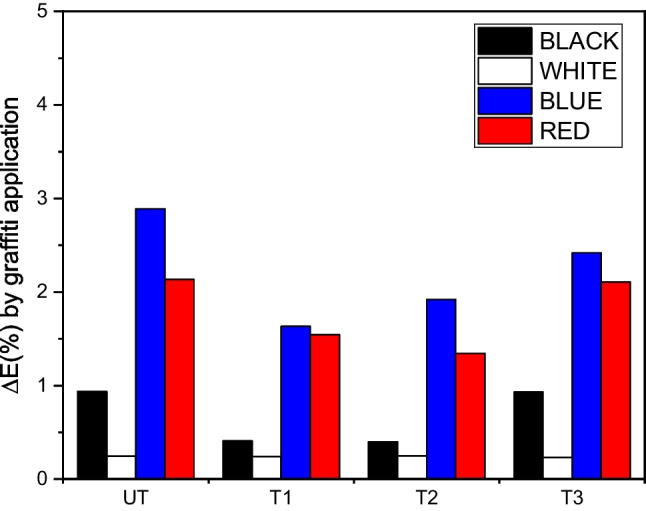
Colour variation of substrate materials, ∆*E*_RGB_ (%) by graffiti paint application on uncoated and TiO_2_- coated slabs

The sample cross-sections (Fig. 10) revealed that the photocatalytic coatings did not prevent the penetration of paint into the substrate. T1 and T2 showed higher penetration than UT and T3, which may be attributed to the hydrophilic behaviour of these coatings (Fig. 8a). Black and white graffiti penetrated to a smaller extent and remained mainly as a layer on the surface. In contrast, the red and blue graffiti penetrated the substrate through microfissures and/or pores in the concrete. However, the colour variation caused by the application of red and blue graffiti paints on the specimen surface (Fig. 11) was greater than that caused by the black and white paints. Given the previous evidence of lower penetration of these paints, this discrepancy can be attributed to the similar chromatic properties of the substrate concrete (light grey) and the black and white paints, which reduces the global change in colour perception. In addition, the surface colour values between TiO_2_ treatments showed that T1 and T2 exhibited lower colour variation owing to their higher penetration properties than UT and T3, in line with previous data.

### Laboratory photocatalytic efficiency

**Fig. 12:**
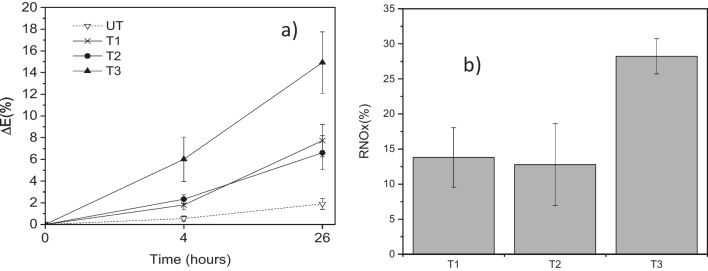
Photocatalytic efficiency of the tested materials. **a** % degradation of RhB dye calculated from the % of global colour change and **b** % of removal of NO_*x*_ gas (RNO_*x*_), specimen surface area of 50 cm.^2^

Figure 12a shows the variation of the global colour change of rhodamine B (RhB) dye after 4 (Δ*E*4) and 26 h (Δ*E*26) of irradiation for all the samples. In the absence of photocatalyst (UT), RhB underwent slight degradation by photolysis. T3 exhibited the highest self-cleaning activity, followed by T1 and T2, whose efficiencies were similar. Similar to the RhB results, the NO_*x*_ air removal percentage (Fig. 12b) showed the highest activity for T3, whereas T1 and T2 exhibited similar lower activities. Overall, T3 appeared to be the most efficient formulation.

### Monitoring the graffiti cleaning efficiency under outdoor exposure conditions

**Fig. 13:**
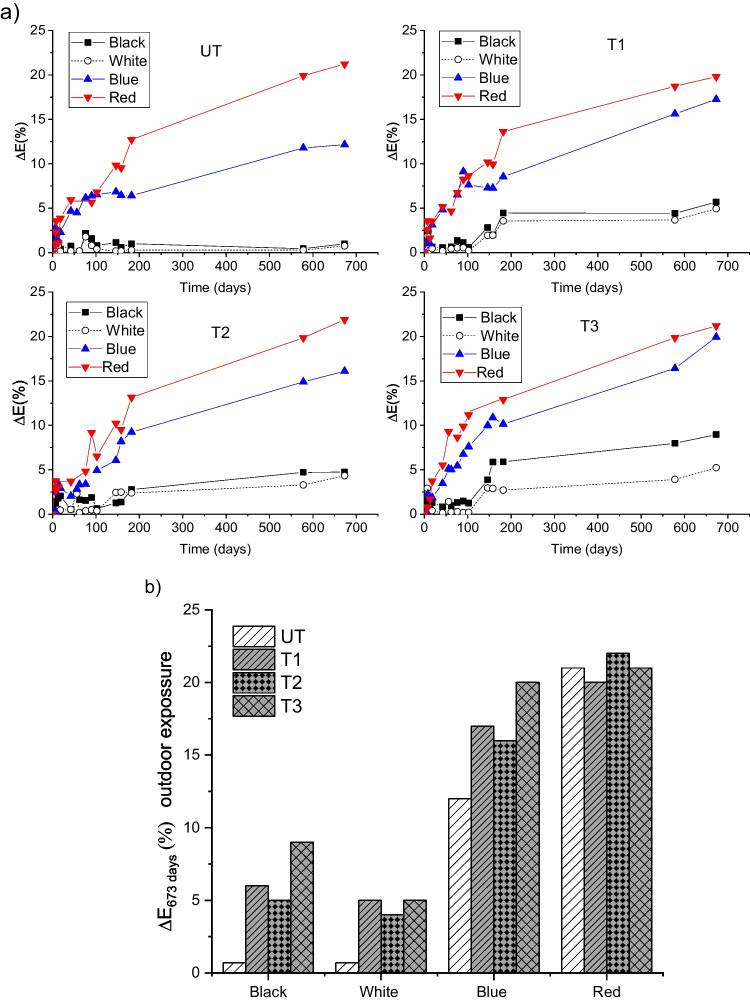
**a** Graffiti colour evolution (∆*E*%) of uncoated and TiO_2_-coated slabs including data corresponding to duplicate slabs, **b** comparison of variation of colour (∆*E*%) after 673 days of outdoor exposure conditions

Figure 13a shows the evolution of graffiti colour (∆*E*, %) in the uncoated and TiO_2_-coated slabs (using the procedure described in Fig. 3). The final ∆*E* after 673 days of outdoor exposure condition is summarised in Fig. 13b. The black and white graffiti did not show significant colour degradation in the untreated slabs; however, a discernible colour variation was observed for the TiO_2_-coated slabs (T3 > T1 > T2), which is consistent with the previous photocatalytic efficiency tests (section “Laboratory photocatalytic efficiency”). In contrast, the blue and red graffiti exhibited significant colour variation upon exposure, even on the untreated slabs. This high degree of colour variation was due to the outdoor ageing effect. The colour variation of the blue graffiti followed the order T3 > T1 > T2 > UT, which again demonstrated the potential of TiO_2_ photocatalytic coatings as a graffiti cleaning procedure. In contrast, the data for the red colour do not show clear evidence that the TiO_2_ treatment improves cleaning effectiveness.

**Fig. 14:**
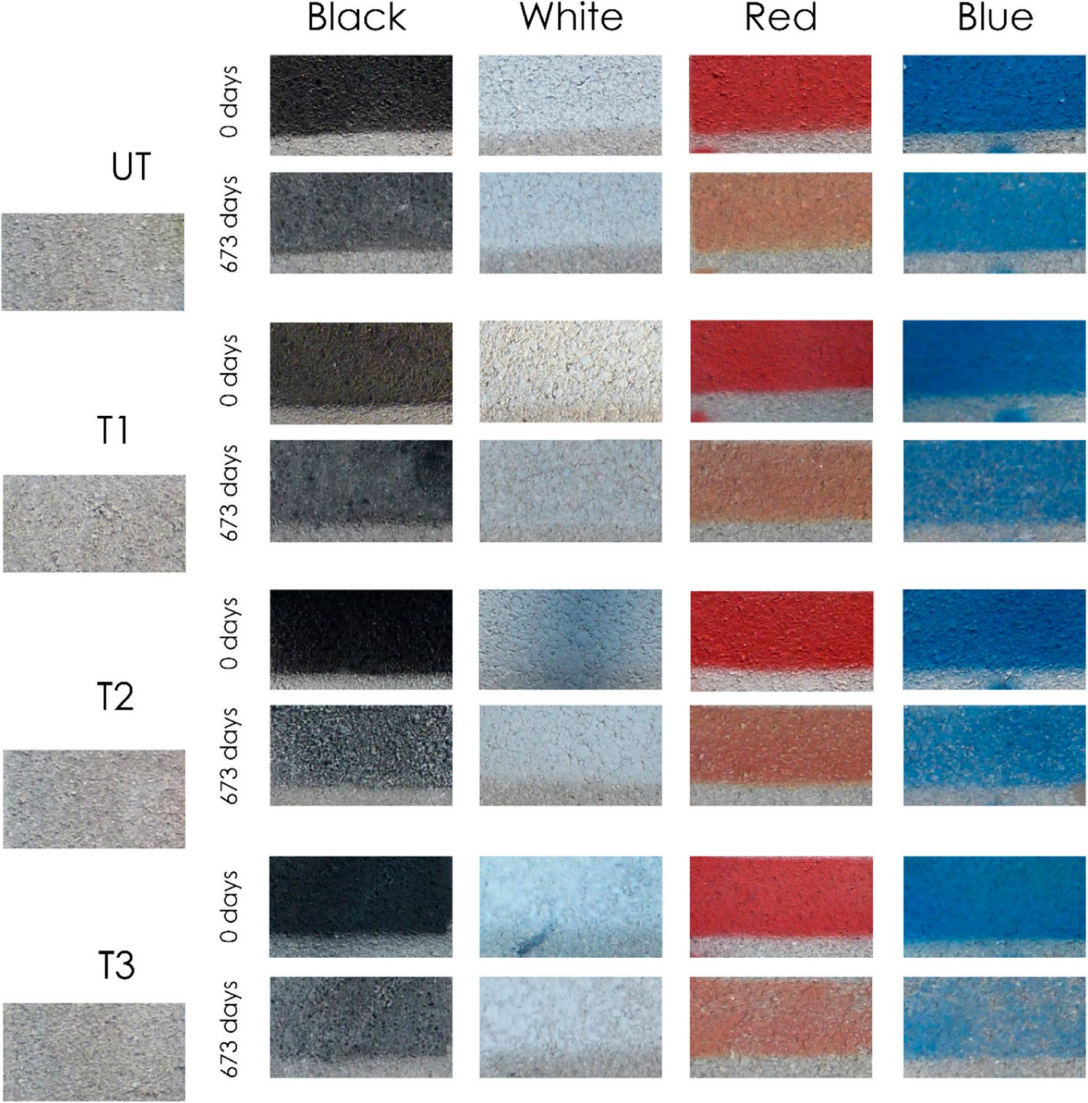
Pictures of the different samples, before painting, immediately after painting, and after 673 days of outdoor exposure

Figure 14 shows a picture of the graffiti when painted and after 673 days of outdoor exposure. The coating in T3 showed the highest cleaning effectiveness, for all colours. The black paint exhibited more colour loss than the calculated data Fig. 12a–b. This is an underestimation of the obtained values because the loss of black colour implies the emergence of the lighter substrate colour (light grey). Nonetheless, it does not invalidate the comparability of cleaning efficacy across samples. In contrast, all the red paints appeared yellowish upon ageing, which was more perceptible in the untreated samples. This may explain the smaller variation of ∆*E* of the red paint the photocatalytic slabs with respect to the untreated sample. This could reduce the reliability of the proposed calculations when the substrate and graffiti paint have similar chromatic properties or when the paint shows a clear colour change. However, considering previous evidence that the application of TiO_2_ coatings does not always provide a protective barrier (section “Characterisation of the graffiti-painted surfaces” and section “Monitoring the graffiti cleaning efficiency under outdoor exposure conditions”), the significantly enhanced observed cleaning performance of the TiO_2_-coated samples is likely related to the photocatalytic redox reactions that decompose the graffiti paint. This can also be related to the hydrophilicity of the TiO_2_ coatings, which may prevent the adhesion and/or penetration of graffiti paint on the surface and/or into the pore matrix.

### Monitoring of the residual colour after anti-graffiti product application and water-pressure cleaning

**Fig. 15:**
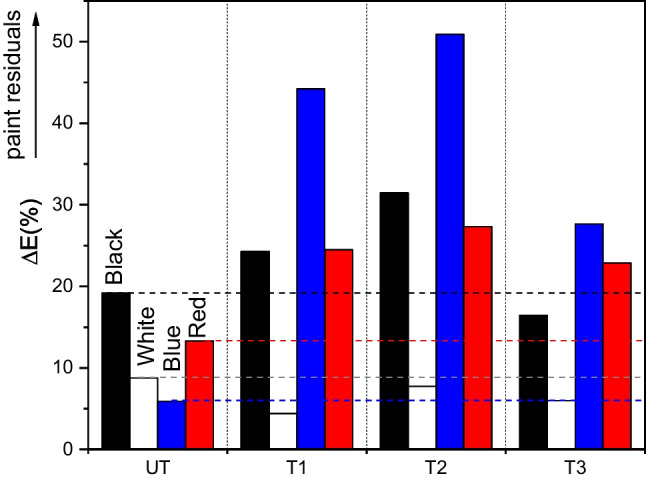
Residual paint calculated from the total colour after the chemical remover application with respect to the original colour of the sample

The ease of the graffiti removal using an anti-graffiti gel-stripper product was quantified by measuring the Δ*E* (%) values between the pictures taken after the anti-graffiti product application and those of the original sample as described earlier (section “Photocatalytic efficiency”, Fig. 15). Although the cleaning procedure was successful, the graffiti paint still remained in every specimen. The untreated slab showed the best results, indicating that the photocatalytic layers did not help to clean the pavement using the traditional method (with solvent and pressurised water). This may be because the TiO_2_ surface layer does not always prevent paint penetration into the substrate (section “Characterisation of the graffiti-painted surfaces”). However, the efficiency of the conventional cleaning operation is directly related to the level of paint penetration in the matrices. In fact, the red and blue graffiti, which show more penetration (section “Characterisation of the graffiti-painted surfaces”), displayed more residual paint after the anti-graffiti product application in the TiO_2_-coated samples than seen in the uncoated sample. In contrast, more residual black graffiti (remaining mainly as a layer on the surface) was seen on T1 and T2, which showed the highest penetration (section “Characterisation of the graffiti-painted surfaces”). For the white paint, the TiO_2_-coating did not seem to modify the cleaning efficiency of the anti-graffiti product. Maximum white residual paint was observed on T2, followed by T1 and T3.

## Conclusions

The aim of this study is to examine the feasibility of using TiO_2_-based coatings on construction materials to obtain self-cleaning surfaces that can reduce the damaging effects of graffiti paints. TiO_2_-coatings were sprayed onto three precast concrete tiles commonly found on streets of Madrid. Laboratory testing of the photocatalytic properties of the tested TiO_2_-based coatings showed that they can effectively photodegrade NO_*x*_ (air pollutant) and RhB (organic dye discolouration). The aesthetic aspect of the treated tiles was only negligibly modified by the TiO_2_-based coatings, well below the perception threshold (ΔE* < 3).

The effectiveness of graffiti cleaning was evaluated onsite by colour measurements over the course of 673 days using four colours of paints (black, white, red, and blue). After 170 days, a chemical remover was used to clean the stained surface to assess the impact of the photocatalytic coatings on a standard cleaning procedure. Although the outcomes were highly influenced by the colour of the graffiti and the properties of the TiO_2_ coatings, it was shown that the transparent TiO_2_-emulsion coatings are promising candidates for treatments to clean graffiti through photo-induced redox reactions that decompose the paint. Beside this, they can prevent adhesion and/or penetration of the graffiti paint on the surface and/or in the pore matrix by their hydrophilicity. However, these coatings do not always serve as physical barriers to prevent graffiti paint from adhering to the surface and increase ease of cleaning with solvents and/or water pressure.
